# Smoking, Radiotherapy, Diabetes and Osteoporosis as Risk Factors for Dental Implant Failure: A Meta-Analysis

**DOI:** 10.1371/journal.pone.0071955

**Published:** 2013-08-05

**Authors:** Hui Chen, Nizhou Liu, Xinchen Xu, Xinhua Qu, Eryi Lu

**Affiliations:** 1 College of Stomatology, Shanghai Jiao Tong University School of Medicine, Shanghai, China; 2 Department of Prosthodontics, Shanghai Ninth People's Hospital, College of Stomatology, Shanghai Jiao Tong University School of Medicine, Shanghai, China; 3 Department of Orthopaedics, Shanghai Key Laboratory of Orthopaedic Implant, Shanghai Ninth People's Hospital, Shanghai Jiao Tong University School of Medicine, Shanghai, China; Iran University of Medical Sciences, Iran (Islamic Republic of)

## Abstract

**Background:**

There are conflicting reports as to the association between smoking, radiotherapy, diabetes and osteoporosis and the risk of dental implant failure. We undertook a meta-analysis to evaluate the association between smoking, radiotherapy, diabetes and osteoporosis and the risk of dental implant failure.

**Methods:**

A comprehensive research on MEDLINE and EMBASE, up to January 2013, was conducted to identify potential studies. References of relevant studies were also searched. Screening, data extraction and quality assessment were conducted independently and in duplicate. A random-effects meta-analysis was used to pool estimates of relative risks (RRs) with 95% confidence intervals (CIs).

**Results:**

A total of 51 studies were identified in this meta-analysis, with more than 40,000 dental implants placed under risk-threatening conditions. The pooled RRs showed a direct association between smoking (n = 33; RR = 1.92; 95% CI, 1.67–2.21) and radiotherapy (n = 16; RR = 2.28; 95% CI, 1.49–3.51) and the risk of dental implant failure, whereas no inverse impact of diabetes (n = 5; RR = 0.90; 95% CI, 0.62–1.32) on the risk of dental implant failure was found. The influence of osteoporosis on the risk of dental implant failure was direct but not significant (n = 4; RR = 1.09; 95% CI, 0.79–1.52). The subgroup analysis indicated no influence of study design, geographical location, length of follow-up, sample size, or mean age of recruited patients.

**Conclusions:**

Smoking and radiotherapy were associated with an increased risk of dental implant failure. The relationship between diabetes and osteoporosis and the risk of implant failure warrant further study.

## Introduction

Dental osseointegrated implants are generally considered as effective and predictable restorations for the replacement of missing teeth. However, although highly desirable outcomes and the long-term survival of dental implant treatments are well documented in numerous studies [1]–[4], implant failures still occur for various reasons. Therefore, the risks associated with dental implant failure have become a frequently discussed topic in recent dental research.

A variety of conditions, including implant design (length, shape or surface texture), patient-related medical risk factors (systemic diseases or habits, such as smoking,), and surgery-related factors (surgeon's experience or surgical design) have been considered to influence the outcome for implant restoration [5]–[7]. With the dramatic advancements in materials science and surgical techniques, increasing attention is focused on patient-related conditions as risk factors for dental implant failure [8].

According to research by Buser and colleagues', patients exposed to with irradiation (radiotherapy) before or after implantation, or patients with severe diabetes or heavy smoking habits have significantly increased risks of dental implant failure [9]. It has been suggested that such conditions could impair implant survivability by increasing the susceptibility of the patient to other diseases or by interfering with the tissue healing process [1]. Moreover, osteoporosis, with its high prevalence in the aged population, is also considered a relative contraindication for dental implant therapy [10], [11]; the alveolar ridge atrophy and low bone mineral density, caused by osteoporosis may impair bone quality and quantity at implant sites [12], [13]. While a number of studies have assessed the influence of smoking, radiotherapy, diabetes and osteoporosis on implant failure, the results have been inconsistent.

Since life expectancy is expected to increase with the advent of better therapies and targeted medicine, an increasing number of patients who smoke or previously smoked, who received radiotherapy for head and neck cancer treatment, or who present with diabetes or osteoporosis may require dental implant treatment. The aim of the present study was, therefore, to provide a comprehensive and critical meta-analysis of clinical studies published in international peer-reviewed literature concerning these four factors of high prevalence and/or of high risks, in order to draw evidence-based conclusions as to the influence of these factors on the outcome of dental implant treatment.

## Methods

### Search Strategy

We performed a systematic literature search of MEDLINE and EMBASE database up to January 2013. All searches were performed using medical subject heading (MeSH) or free text words. We combined search terms for outcomes (survival, success, osseointegration, failure, removal, replacement and loss), risk factors (1, smoking, smoker or tobacco; 2, irradiation, radiotherapy or head and neck cancer; 3, diabetes, diabetic, diabetes mellitus or hyperglycemia; 4, osteoporosis, osteopenia, low bone mineral density or bone loss) and key subjects (dental implant or oral implant). Reference lists of identified articles and relevant papers known to reviewers were also searched. Emails were sent to the authors of identified studies for additional information, where necessary. Studies were limited to English publications. Considering the study by Mish and his colleagues, we referred implant removal or implant loss to “implant failure” [14].

### Selection Criteria

Three reviewers (H Chen, N Liu and X Xu) conducted the search independently. Titles and abstracts were screened for subject relevance. Studies that could not be definitely excluded based on abstract information were also selected for full text screening. Two reviewers examined the full text of all relevant studies for inclusion possibility (smoking: N Liu and X Xu; radiotherapy: H Chen and X Xu; diabetes and osteoporosis: H Chen and N Liu). Where there was a disagreement for study inclusion, a discussion was held with a third reviewer (X Qu) to reach a consensus.

Studies were eligible for inclusion if they met the following criteria: (1) human study; (2) observational study; (3) studies focusing on the influence of smoking and/or radiotherapy and/or diabetes and/or osteoporosis on dental implant failure; (4) studies providing outcome data for dental implant failure or relevant data that could be calculated by the reviewers; (5) studies providing data for both a non-risk (control) group and a risk (study) group; (6) studies published in English. Exclusion criteria were agreed as follows: (1) animal study; (2) in vitro or laboratory study; (3) review or case report; (4) studies providing craniofacial implant data for which dental implant data could not be extracted; (5) studies providing patient-related data (to be specific, survival/failure rate that was calculated at the patient-level); 6) studies without data on non-smoking, non-irradiation, non-diabetic or non-osteoporotic groups.

### Data Extraction and Quality Assessment

Two reviewers (H Chen and N Liu) independently extracted data using a structured form. The following information was extracted from each included study: year of publication, country, first author's family name, study design, follow-up period, characteristics of subjects (number of patients, gender and age), information relevant to risk factors, characteristics of the dental implants (number and placement position) and data on dental implant failure.

The methodological quality of the included studies was independently and appraised twice by two reviewers (H Chen and X Xu) using elements of McMaster Quality Assessment Scale of Harms (McHarm) [15]. The criteria of the quality assessment are presented in Table 1. Any discrepancy that occurred during data extraction and quality assessment was resolved by consensus or discussion with another reviewer (X Qu).

**Table 1:** Criteria of Quality Assessment (a Modified McHarm checklist).

	ITEMS	YES	NO/Not sure
**1**	**Were the harms PRE-DEFINED using standardized or precise definitions?**	**1**	**0**
	(In present study, we defined “harms” as the totality of adverse consequences of an implant surgery)		
**2**	**Were SERIOUS events precisely defined?**	**1**	**0**
	(In present study, we defined complications that didn't lead to IMPLANT LOSS or IMPLANT REMOVAL as SERIOUS events, e.g. sensitivity on function, radiographic bone loss ≤4 mm or 1/2 of the implant body, probing depth ≤7 mm, etc. [14])		
**3**	**Were SEVER events precisely defined?**	**1**	**0**
	*(In present study, we defined IIMPLANT LOSS as SERIOUS events)*		
**4**	**Did the study specify the TRAINING or BACKGROUND of who ascertained the harms?**	**1**	**0**
**5**	**Did the study specify the TIMING and FREQUENCY of collection of the harms?**	**1**	**0**
**6**	**Did the author(s) use STANDARD scale(s) or checklist(s) for harms collection?**	**1**	**0**
**7**	**Was the NUMBER of participants that withdrew or were lost to follow-up specified for each study group?**	**1**	**0**
**8**	**Was the TOTAL NUMBER of participants affected by harms specified for each study arm?**	**1**	**0**
**9**	**Did the author(s) specify the NUMBER for each TYPE of harmful event for each study group?**	**1**	**0**
**10**	**Did the author(s) specify the type of analyses undertaken for harms data?**	**1**	**0**
**A Total of 10 Points**			

### Statistical Analysis

We evaluated dental implant failure for any reason attributable to the implant as our outcome measure of interest. Relative risk (RR) was used as the common measure of association across studies. The RRs and 95% confidence intervals (CIs) were extracted or calculated from each study, and then we pooled the overall RRs using the inverse of corresponding variances as weights. For the meta-analysis, a random-effects model was considered [16]. Heterogeneity between studies was tested through the Cochran Q and *I* ^2^ statistics (*I* ^2^ values of 25, 50, and 75% are considered as low, moderate, and high, respectively [17]).

Subgroup analyses were used to identify associations between the risk of dental implant failure and other relevant study characteristics (mean age, geographical location, design of study, sample size and length of follow-up) as possible sources of heterogeneity. Publication bias was measured using Begg's and Egger's regression tests and visualization of funnel plots [18]. The stability of the study was also detected by sensitivity analysis, through re-meta-analysis with one involved study excluded each time. All statistical analyses were performed with Review Manager 5.01 (The Cochrane Collaboration, Copenhagen, Denmark) and Stata version 11 (StataCorp, College Station, TX).

## Results

### Literature Search

**Figure 1:**
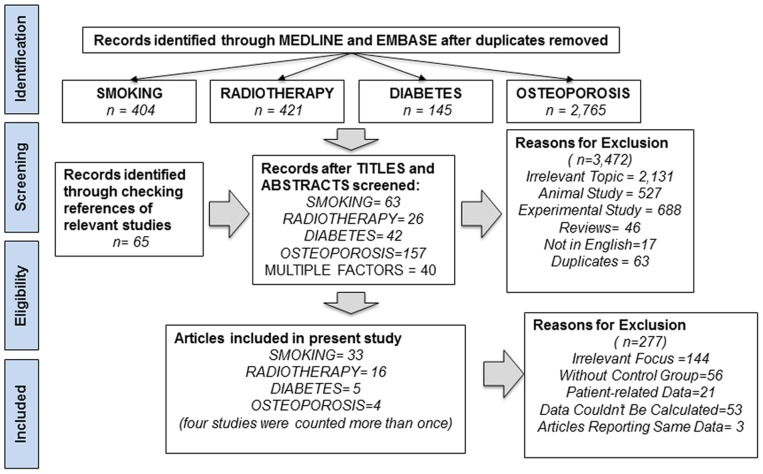
Flow Diagram of Screened and Included Papers.

The literature search yielded a total of 3,735 primary studies, of which 3,472 were excluded after title screening. An additional 65 studies were included after checking the references of relevant reviews and studies. Finally, 328 studies were included for full-text assessment, of which 277 were excluded for one of the following reasons: (1) studies focusing on irrelevant outcome assessment (n = 144), such as bone loss or primary stability; (2) studies without a non-risk group (n = 56); (3) studies only providing patient-related data (n = 21); (4) studies where data related to implant failure could not be calculated (n = 53); and (5) studies where the reported data were represented in another included in our analysis (n = 3) [19]–[21]. As a result, 51 studies met the inclusion criteria for meta-analysis, with 33 studies for smoking [22]–[54], 16 for radiotherapy [31], [44], [55]–[68], five for diabetes [31], [44], [47], [48], [69] and four for osteoporosis [44], [70]–[72], respectively. Of note, four studies involved more than one risk factor and were included in more than one group [31], [44], [47], [48]. A flow diagram of the study selection process is presented in Figure 1.

### Study Characteristics and Quality Assessment

The detailed characteristics of the included studies and the results of the quality assessment are summarized in Tables 2–5. The number of implants in each study ranged from 56 [34] to 5,843 [49]. The earliest study was published in 1993 [22], and the latest in 2012 [53], [54], [67]–[69]. In terms of study design, 23 studies enrolled patients prospectively [24], [25], [27]–[29], [32]–[34], [37]–[40], [42], [46], [47], [55], [58], [60]–[62], [64], [66], [69], whereas 28 were retrospective database reviews [22,23,26,30,31,35,36,41,43–45,48–54,56,57,59,63,65,67,68,70–72]. By geographic location, 18 studies were conducted in the United States [24,26,30–33,35,36,38–40,42,45,50,53,57,65,71], 24 in Europe [23,27,28,29,37,43,44,49,51,52,54–56,58–64,66–68,72] and nine in other regions [22,25,34,41,46–48,69,70]. The overall study quality averaged 8.2 (range, 5–10) on a scale of 1 to 10.

**Table 2:** Study Characteristics (SMOKING).

Author (Year)	Country	Study	Follow-up	Patient Characteristics			Smoking	Implant Characteristics			QS
				Mean Age	CON/STY	F		CON/STY	Position (Mand./Max.)	FC/FS	
Bain , 1993	Canada	Retro	37.88 m	54.7 yr	NA/NA	311	NA	1,804/390	1,115/1,079	86/44	8
De Bruyn, 1994	Belgium	Retro	NA	(20–80 yr)	91/26	66	NA	338/114	208/244	5/10	8
Gorman, 1994	USA	Prospec	NA	NA	228/82	NA	NA	142/646	NA	47/42	7
Bain, 1996	Canada	Prospec	NA	NA	NA/NA	NA	NA	176/47	NA	10/9	8
Minsk, 1996	USA	Retro	6 yr	NA	NA/NA	NA	20 per day	570/157	358/369	52/17	9
Lindquist, 1997	Sweden	Prospec	10 yr	(33–64 yr)	24/21	32	NA	139/125	Mandible	3/0	8
De Bruyn, 1999	Belgium	Prospec	7 yrs	NA	13/10	NA	13.2 per day	32/30	Maxilla	9/6	10
Grunder, 1999	Switzerland	Prospec	34.4 m	58±15 yr	55/19	34	NA	164/55	NA	3/0	9
Jones, 1999	USA	Retro	58 m	50 yr	44/19	40	NA	217/126	204/147	5/11	8
Keller, 1999	USA	Retro	12 yr	(15–73 yr)	26/28	NA	NA	143/105	Grafted maxilla sinus	26/7	10
Lambert, 2000	USA	Prospec	3 yr	NA	NA/NA	NA	NA	1,928/959	1616/1271	115/85	8
Olson, 2000	USA	Prospec	38±15 m	56±12 yr	NA/NA	1	NA	65/51	Grafted maxillary sinus	1/2	7
Wallace, 2000	USA	Retro	4 yr	NA	39/17	27	NA	115/72	NA	8/12	7
Schwartz-Ara, 1999	Israel	Prospec	5 yr	47 yr	NA/NA	27	NA	50/6	39/17	5/1	7
Geurs, 2001	USA	Retro	3.2±1.3 yr	NA	NA/NA	NA	NA	267/62	Grafted maxilla sinus	13/7	6
Widmark, 20001	Sweden	Prospec	(3–5 yr)	NA	25/11	NA	≥half a pack a day	131/67	Local: 120/Grafted: 101	14/26	10
Kumar, 2002	USA	Prospec	NA	NA	389/72	NA	NA	914/269	357/826	8/15	5
Van Steenberghe, 2002	Belgium	Prospec	NA	50±14 yr	NA/NA	243	NA	1,107/156	NA	19/8	7
Karoussis, 2003	Switzerland	Prospec	10 yr	NA	41/12	NA	NA	84/28	NA	3/2	10
DeLuca, 2006	Canada	Retro	59.8 m	49.3 yr	285/104	283	NA	1,045/4,94	NA	32/26	9
Peleg, 2006	USA	Prospec	69 m	NA	505/226	453	NA	1,505/627	Maxilla sinus grafting	28/16	7
Mundt, 2006	Germany	Retro	88.2 m	54.1 yr	NA/NA	94	NA	294/363	296/367	6/30	8
Alsaadi, 2008	Belgium	Retro	2 yr	NA	351/61	240	NA	1,291/223	698/816	80/21	8
Balshe, 2008	USA	Retro	5 yr	49.4 yr	1299/119	861	17.7±7 per day	3,841/766	2,633/1974	188/77	7
Levin, 2008	Israel	Prospec	6.14 yr	45 yr	54/10	40	NA	54/10	NA	3/1	7
Tawil, 2008	Lebanon	Prospec	42.4 m	NA	50/40	33	NA	254/245	NA	2/5	9
Anner, 2010	Isreal	Retro	31±28 m	52±12 yrs	412/63	299	NA	1,400/226	NA	56/21	7
Cavalcanti, 2011	Italy	Retro	5 yr	50 yrs	1019/458	1025	NA	3,882/1,961	NA	112/107	9
Conrad, 2011	USA	Retro	35.7 m	55.3 yr	NA/NA	168	NA	446/48	Maxilla	28/6	8
Rodriguez, 2011	Spain	Retro	≥6 m	53±13 yr	182/113	188	NA	644/389	NA	18/14	9
Vandeweghe, 2011	Belgium	Retro	22 m	54±13.4 yr	288/41	43	NA	608/104	NA	7/5	9
Lin, 2012	USA	Retro	12 m	59.6 yr	47/28	186	NA	93/62	Grafted maxiila sinus	12/13	9
Vervaeke, 2012	Belgium	Retro	31±7.2 m	56±12 yr	235/60	168	NA	244/849	458/648	11/8	10

CON = control group, that is non-smoking group;STY = study group, that is smoking group; F = Female; Mand. = mandible; Max. = maxilla; Retro = retrospective study; Prospec = prospective study; yr = year; m = month; NA = not available; Local = local bone; Grafted = grafted bone; FC = failure implant number of Control Group; FS = failure implant number of Study Group; QS = quality assessment score.

**Table 3:** Study Characteristics (RADIOTHERAPY).

Author (Year)	Country	Study	Follow-up	Patient Characteristics			Radiotherapy		Implant Characteristics			QS
				Mean Age	CON/STY	F	Time	Dose (Gy)	CON/STY	Position (Mand./Max.)	FC/FS	
Esser, 1997	Germany	Prospec	NA	(37–79 yr)	NA/NA	9	BP	60	66/152	Mandible	7/33	7
Werkmeister, 1999	Germany	Retro	3 yrs	55 yr	17/12	6	BP	54	79/30	Local: 64/Grafted: 45	19/8	7
Keller, 1999	USA	Retro	12 yrs	(15–73 yr)	52/2	NA	NA	55 and 61	237/11	Grafted maxilla	33/0	10
Shaw, 2005	UK	Retro	3.5 yr	58 yr	43/34	32	BP	40–66	192/172	Local: 238/Grafted: 126	25/31	9
Yerit, 2006	Austria	Prospec	5.4±3.2 yr	58±14 yr	NA/NA	15	BP	50	162/154	Local: 238/Grafted: 78	15/29	9
Schepers, 2006	Netherlands	Retro	up to 23 m	66.11 yr	27/21	19	AP	60–68	78/61	NA	0/2	8
Landes, 2006	Germany	Prospec	36 m	63 yr	11/19	8	BP	57	42/72	NA	0/1	8
Nelson, 2007	Germany	Prospec	10.3 yr	59 yr	NA/29	30	BP	up to 72	311/124	281/154	4/7	7
Alsaadi, 2008	Belgium	Retro	2 yr	NA	410/2	240	NA	NA	1,499/15	698/816	98/3	8
Schoen, 2008	Netherlands	Prospec	12 m	62±11 yr	16/19	15	AP	60.1±7.7	64/76	Local bone	2/2	9
Klein, 2009	Germany	Retro	5 yr	58.4 yr	16/27	12	BP	<50 or ≥50	74/116	Local: 62/Grafted: 128	12/13	8
Cuesta-Gil, 2009	Spain	Prospec	/	52 yr	32/79	31	Mixed	50–60	311/395	Local: 454/Grafted: 252	6/75	9
Salinas, 2010	USA	Retro	41.1	NA	18/26	19	Mixed	> 60	116/90	Local: 105/Flap: 114	8/23	10
Linsen, 2012	Germany	Prospec	48±34.3 m	56±16 yr	32/34	23	BP	36 or 60	135/127	213/49	6/8	10
Jacobsen, 2012	Switzerland	Retro	67 m	52.4 yr	NA/NA	16	AP	NA	93/47	Local: 41/Flap: 99	14/14	9
Fenlon, 2012	UK	Retro	/	NA	29/12	NA	AP	66	110/35	Grafted bone	3/15	8

CON = control group,that is non-radiotherapy group; STY = study group, that is radiotherapy group; F = Female; BP = before placement; AP = after placement; Mand. = mandible; Max. = maxilla; Retro = retrospective study; Prospec = prospective study; yr = year; m = month; NA = not available,; Local = local bone; Grafted = grafted bone; FC = failure implant number of Control Group; FS = failure implant number of Study Group; QS = quality assessment score.

**Table 4:** Study Characteristics (DIABETES).

Author (Year)	Country	Study	Follow-up	Patient Characteristics			Diabetes Type	Implant Characteristics			QS
				Mean Age	CON/STY	F		CON/STY	Position (Mand./Max.)	FC/FS	
Keller, 1999	USA	Prosp	12 yrs	(15–73 yr)	52/2	NA	NA	237/11	Grafted maxilla	0/0	10
Morris, 2000	New Zealand	Prosp	36 m	NA	408/255	NA	II	2632/255	Mixed	180/20	7
Tawil, 2008	Lebanon	Retro	42.4 m	62.15 yr	45/45	33F	II	244/255	Mixed	2/7	9
Alsaadi, 2008	Belgium	Retro	2 yr	NA	402/10	240	I:1 II:9	1,480/34	698/816	202/0	8
Anner, 2010	Isreal	Prosp	31±28 m	52±12 yr	426/49	299	NA	1,449/177	Mixed	72/5	7

CON = control group, that is non-diabetes group; STY = study group, that is diabetes group; F = Female; Mand. = mandible; Max. = maxilla; Retro = retrospective study; Prospec = prospective study; yr = year; m = month; NA = not available; Local = local bone; Grafted = grafted bone; FC = failure implant number of Control Group; FS = failure implant number of Study Group; QS = quality assessment score.

**Table 5:** Study Characteristics (OSTEOPOROSIS).

Author (Year)	Country	Study	Follow-up	Patient Characteristics			Implant Characteristics			QS
				Mean Age	CON/STY	F	CON/STY	Position (Mand./Max.)	FC/FS	
Amorim,2007	Brazil	Retro	9 m	58.2 yr	20/19	39	43/39	Mandible	0/1	8
Alsaadi,2008	Belgium	Retro	2 yr	NA	393/19	240	1,446/68	698/816	92/9	8
Holahan,2008	USA	Retro	5.4 yr	63±9 yr	564/192	746	306/340	378/268	17/20	7
Dvorak,2011	Austria	Retro	6±4 yr	≥45 yr	115/62	117	543/258	396/432	17/20	7

CON = control group, that is non-osteoporosis group; STY = study group, that is osteoporosis group; F = Female; Mand. = mandible; Max. = maxilla; Retro = retrospective study; Prospec = prospective study; yr = year; m = month; NA = not available; Local = local bone; Grafted = grafted bone; FC = failure implant number of Control Group; FS = failure implant number of Study Group; QS = quality assessment score.

### Smoking

**Figure 2:**
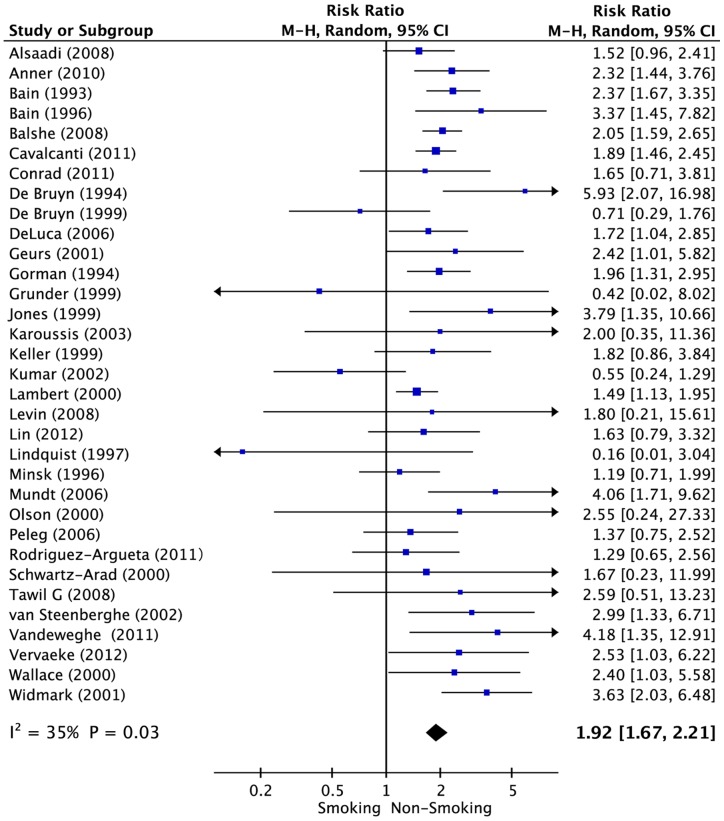
Forest plot of studies with dental implant failure risk for smoking versus non-smoking patients. The combined Relative risks (RR) and 95% confidence intervals (CIs) were calculated using the random-effects model.

The multivariable-adjusted RRs in each study and the pooled RRs of dental implant failure for smoking versus non-smoking patients are presented in Figure 2, Table 2 and Table 6 (33 studies; 35,118 implants). In the pooled analysis, smoking was associated with higher risk of dental implant failure (RR = 1.92; 95% CI, 1.67–2.21). There was moderate heterogeneity among the studies (*P* = 0.03, *I^2^* = 35%). Stratifying by study design, the pooled RRs for prospective studies and retrospective studies were 1.34 (95% CI, 0.90–2.00) and 2.01 (95% CI, 1.75–2.30). Stratifying by geographical location, the summary RRs were 1.59 (95% CI, 1.27–1.98) for studies conducted in the United States, 2.27 (95% CI, 1.62–3.20) for Europe and 2.23 (95% CI, 1.77–2.81) for other regions. With regard to the mean age of patients, the pooled RRs for <55-year-old and ≥55-year-old patients were 2.15 (95% CI, 1.87–2.47) and 1.67 (95% CI, 1.13–2.47), respectively. A subgroup analysis indicated no influence of study design, geographical location, length of follow-up, sample size or mean patient age.

**Table 6:** Subgroup analysis to investigate differences between studies included in meta-analysis.

Subgroup	No. of Studies	RR (95% CI)	*I* ^2^ (%)	*P* value	*P* value for heterogeneity between subgroups
**Smoking**					
**Design of Study**					
Prospective	15	1.34(0.90,2.00)	67	<0.0001	0.06
Retrospective	18	2.01(1.75,2.30)	14	0.29	
**Geographical Location**					
United States	13	1.59 (1.27,1.98)	46	0.04	0.08
Europe	13	2.18 (1.56,3.05)	56	0.007	
Other Regions	7	2.23 (1.77,2.81)	0	0.90	
**Length of Follow-up (years)**					
≥5	11	1.72 (1.37,2.15)	28	0.18	0.32
<5	17	1.98 (1.68,2.33)	14	0.29	
**Sample Size (implant)**					
<500	16	2.25 (1.64,3.08)	25	0.17	0.23
≥500	17	1.81 (1.56,2.11)	40	0.05	
**Age (years)**					
<55	11	2.15 (1.87,2.47)	0	0.67	0.23
≥55	6	1.67 (1.13,2.47)	0	0.54	
**Radiotherapy**					
**Design of Study**					
Prospective	6	2.02 (1.37,2.97)	0	0.73	0.58
Retrospective	10	2.50 (1.32,4.75)	81	<0.00001	
**Geographical Location**					
United States	2	1.46 (0.12,17.16)	69	0.07	0.72
Europe	14	2.29 (1.45,3.63)	71	<0.0001	
**Length of Follow-up (years)**					
≥5	5	1.62 (0.85,3.11)	62	0.03	0.83
<5	8	1.76 (1.20,2.59)	20	0.27	
**Sample Size (implant)**					
<250	10	2.14 (1.27,3.60)	64	0.003	0.56
≥250	6	2.74 (1.43,5.25)	76	0.001	
**Age (years)**					
<60	8	1.95 (1.11,3.42)	78	<0.0001	0.68
≥60	3	1.40 (0.33,5.97)	0	0.69	

### Radiotherapy

**Figure 3:**
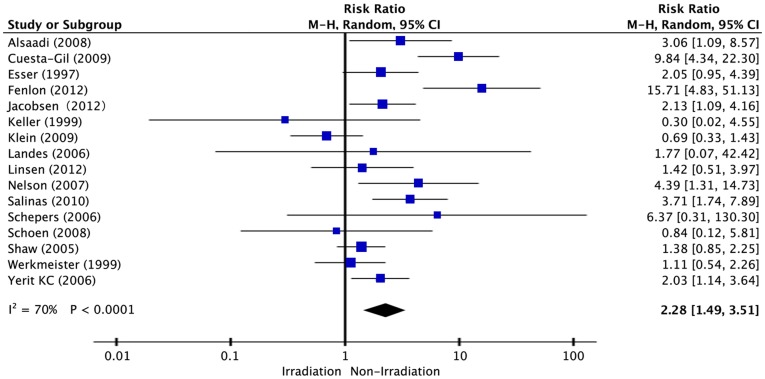
Forest plot of studies with dental implant failure risk for patients with radiotherapy versus non-smoking. The combined Relative risks (RR) and 95% confidence intervals (CIs) were calculated using the random-effects model.

Figure 3 shows the association between radiotherapy and risk of dental implant failure from a collection of 16 studies and 5,246 implants. A pooled analysis indicated a direct association between radiotherapy and the risk of dental implant failure (RR = 2.28; 95% CI, 1.49–3.51). The heterogeneity among the studies was high (*P*<0.0001, *I^2^* = 70%). As far as geographical location was concerned, the summary RRs were 1.46 (95% CI, 0.12–17.3) for studies performed in the United States and 2.29 (95% CI, 1.45–3.63) for Europe. Stratifying by length of follow-up, the pooled RRs for <5-year and ≥5-year duration were 1.76 (95% CI, 1.20–2.59) and 1.62 (95% CI, 0.85–3.11), respectively. According to the mean age of the patients involved, the pooled RRs for <55-year-old and ≥55-year-old patients were 1.95 (95% CI, 1.11–3.42) and 1.40 (95% CI, 0.33–5.97). In the subgroup analysis, study design, geographical location, length of follow-up, sample size and mean patient age, had no influence on the risk of dental implant failure (Table 6).

### Diabetes and Osteoporosis

**Figure 4:**
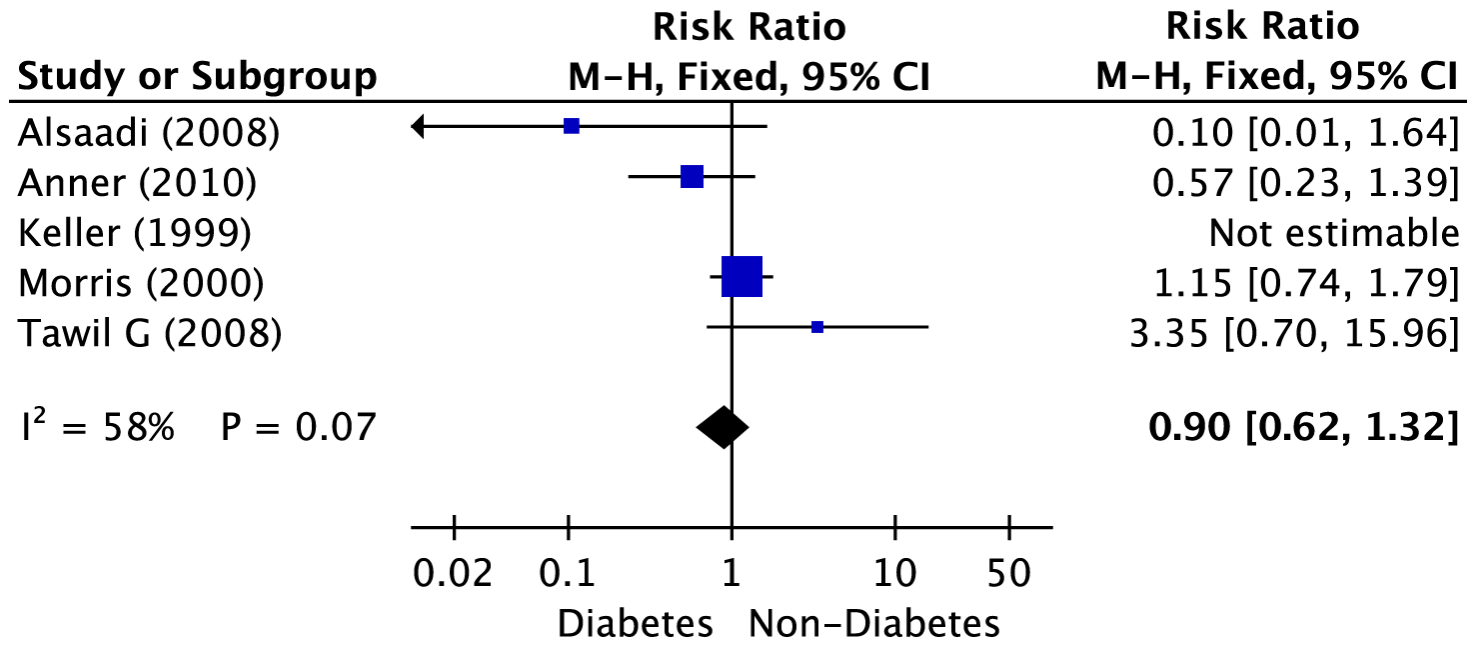
Forest plot of studies with dental implant failure risk for patients with diabetes versus non-diabetes. The combined Relative risks (RR) and 95% confidence intervals (CIs) were calculated using the random-effects model.

Five studies were included to analyze on dental implant failure with regard to diabetes (6,774 implants). The results of the pooled analysis are shown in Figure 4. The pooled RR for patients with diabetes versus patients without diabetes was 0.90 (95% CI, 0.62–1.32), indicating no association between diabetes and the risk of dental implant failure. We found high heterogeneity across the studies (*P* = 0.07, *I^2^* = 58%).

**Figure 5:**
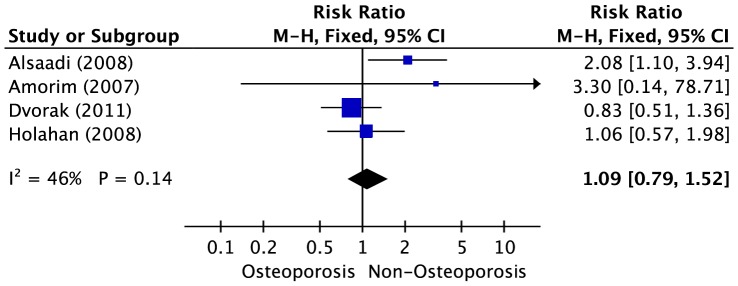
Forest plot of studies with dental implant failure risk for patients with osteoporosis versus non-osteoporosis. The combined Relative risks (RR) and 95% confidence intervals (CIs) were calculated using the random-effects model.

Four studies were concerned with the association between osteoporosis and dental implant failure, with a collection of 3,070 implants. In the pooled analysis, the association between osteoporosis and the risk of dental implant failure was direct but not significant (RR = 1.09; 95% CI, 0.79–1.52), with high heterogeneity across the studies (*P* = 0.14, *I^2^* = 46%). (Figure 5)

Since limited studies focusing on diabetes and osteoporosis met our inclusion criteria, and insufficient data could be extracted from the included studies, no subgroup analysis was performed to further investigate the association between diabetes and osteoporosis and risk of dental implant failure.

### Publication Bias and Sensitivity Analysis

Publication bias was determined by visualization of funnel plot, Begg's test, and Egger's regression test. With the exception of radiotherapy (Begg's test: *P* = 0.47; Egger's test: *P* = 0.02), there was no evidence of publication bias for smoking (Begg's test: *P* = 0.49; Egger's test: *P* = 0.94), diabetes (Begg's test: *P* = 0.33; Egger's test: *P* = 0.23) or osteoporosis (Begg's test: *P* = 0.17; Egger's test: *P* = 0.34). Sensitivity analysis showed that excluding any one study from the pooled analysis did not vary the results substantially. (See Figure S1 for funnel plots of smoking, radiotherapy, diabetes and osteoporosis risk factor)

## Discussion

### Principle Findings

After reviewing numerous studies assessing the potential risk factors for dental implant failure, this meta-analysis supports the view that smoking and radiotherapy are associated with a higher risk of dental implant failure. Our findings suggest that individuals who smoke, or who have undergone radiotherapy before or after implantation, might suffer an approximately 35 or 70% higher risk of dental implant failure, respectively, as compared with non-smokers or those who have not been exposed to radiotherapy. We found no significant inverse impact of diabetes on the risk of dental implant failure, whereas osteoporosis showed a direct but not significant association. However, because of the limited number of studies focusing on diabetes and osteoporosis, these results should be interpreted carefully and verified by further studies. The findings of this meta-analysis, may offer clinical dentists with additional insights into the prognosis of dental implant treatment and may help in the establishment of potential treatment plans.

### Implications

The outcome of this meta-analysis indicated that individuals who smoke were more likely to suffer from dental implant failure. This finding is consistent with a previous meta-analysis performed in 2006, with an elevated OR of 2.17 (95% CI, 1.67–2.83) indicating the inverse impact of smoking on implant osseointegration [73]. Although the underlying mechanism is still not completely understood, researchers previously posited that smoking impaired the wound healing processes involved with implant/tissue integration [27]. Others suggested that smokers treated with implants had an increased risk of postoperative complications, such as infection and peri-implantitis [53]. Bain and colleagues recommended that patients commence a smoking cessation protocol at least one week before and at least two months after dental implant surgery to assure dental implant osseointegration [22]; however, others have demonstrated that pre-operative smoking cessation, especially short-term cessation, bears no significant effect on reducing the risk of dental implant failure [74].

The present meta-analysis indicates that radiotherapy was strongly associated with increased risk of dental implant failure. A former review of animal and human studies reached a similar conclusion that implants placed in irradiated bone experienced 2–3 times higher rates of failure [75]. Moreover, implants placed in irradiated maxilla were reported to have a higher failure rate compared with those in irradiated mandible [76]. Bone responds to irradiation with various cellular, vascular, and metabolic alterations occurring at different sites in the irradiated bone and adjacent tissues [77]. Several plausible mechanisms to explain how bone responds to irradiation have been proposed, including altered osteoblast and osteoclast function during bone repair and remodeling, the formation and the subsequent breakdown of hypoxic-hypocellular and hypovascular tissues, and a decreased rate of tissue perfusion and tissue fibrosis [77–79]. Such responses were previously believed to be highly variable and partly related to the administered dose of radiation [77]. Researchers suggested that a fractionated dose would be better tolerated than a single exposure at the same level of intensity [80]. Furthermore, adjunctive treatment with the use of hyperbaric oxygen (HBO) was expected to increase the regenerative capacity of tissue damaged after radiotherapy; however, no strong evidence was found to support the use of HBO to decrease dental implant failure for radiotherapy-exposed patients [81].

Diabetes and osteoporosis are both highly prevalent disorders among elderly patients [10,11,82]. After reviewing the published literature, we found a lack of high quality and single-risk-factor focused studies with regard to the effects of diabetes or osteoporosis on dental implant survival. The present meta-analysis revealed no direct impact of diabetes or osteoporosis on the risk of dental implant failure, although both were reported to affect wound healing in oral tissues [1]. Clinical dentists are advised to avoid dental implant treatment in poorly controlled diabetic patients, and studies indicate that the long-term use of bisphosphonates by osteoporotic patients may cause osteonecrosis of the jaw [83]. Unfortunately, data was insufficient yet to give an explicit explanation of its effect on risk of dental implant failure. Diabetes and osteoporosis can be well controlled by drug intervention; yet since, none of the studies included a discussion as to the different level of severity of diabetes or osteoporosis in these patients and on the risk of dental implant failure, this limited our ability to further assess the risk of these two factors in the present meta-analysis.

### Strength and Limitations

To our knowledge, this study is the most comprehensive meta-analysis to estimate the association of smoking, radiotherapy, diabetes, and osteoporosis with dental implant failure. We were able to include a substantial total number of subjects (more than 40,000 dental implants placed under risk-threatening conditions), which significantly increased the statistical power of our analysis. We made sure to minimize the bias by means of study procedure. Not only did we search MEDLINE and EMBASE databases to identify potential studies, but also we manually examined all reference lists from relevant studies. The McHarm quality assessment tool was used to evaluate each of the included studies to ensure sufficient study quality (mean score of 8.2 out of 10). Publication bias was also absent, as determined by visualization of funnel plot, Begg's test and Egger's test.

Despite the above strengths and advantages, this meta-analysis has several limitations. First, the present study was subject to confounding factors that could be inherent in the included studies and it is difficult to completely rule out the possibility that other risk factors were responsible for the observed associations. Second, heterogeneity might have been introduced by methodological differences among the studies. Many of the *I^2^* estimates calculated in this meta-analysis were judged as high. While we were able to perform subgroup analyses on studies of smoking and radiotherapy, which indicated no influence on the study design, geographical location, length of follow-up, sample size and mean patient age, the diabetes and osteoporosis implant failure data were insufficient for a stratified analysis. Although these issues might have reduced the strength of the conclusions drawn in this meta-analysis, visual inspection of the forest plots suggests that there is considerable consistency in the RRs across the studies. Third, the search was limited to English studies and only performed with the use of two electronic databases, mainly because of the limited work force for the present research; this might have introduced a selection bias to the results.

### Suggestion for Future Studies

On the basis of this meta-analysis, several questions should be answered in future studies. First, what is the compound effect of multiple risk factors on dental implant failure? For instance, what is the risk of dental implant failure for smokers with diabetes, or smokers with osteoporosis? To answer this question, several well-designed cohort studies with adequate control for confounding factors should be considered. Second, could different severity levels of the four risk factors, such as the severity of the disease or the frequency of smoking, have an effect on dental implant failure? An investigation that specifically focuses on the quantity of smoking, the overall irradiation dose, and/or the severity of diabetes and osteoporosis may offer insight into this question. Third, could the application of smoking cessation or HBO treatment as an adjunct to radiotherapy decrease the risk of dental implant failure? Future studies, including randomized controlled trials, concerning the topics are needed to gain a better understanding of the underlying relationship among these risk factors.

## Conclusions

The present study investigated the influence of smoking, radiotherapy, diabetes and osteoporosis on dental implant failure, and may provide clinical dentists with additional insight for dental implant treatment prognosis and treatment strategies. We found that, smoking and radiotherapy are associated with a higher risk of dental implant failure while diabetes has no significant inverse impact on the risk of dental implant failure. The association between osteoporosis and the risk of dental implant failure was direct but not significant. However, because of the lack of high quality and individual risk-isolated studies with respect to diabetes and osteoporosis, additional, well-designed studies, with adequate control for confounding factors, are required in future investigations.

## Supporting Information

Checklist S1 **PRISMA Checklist. (PDF)**
(PDF)

Figure S1 **Funnel Plot of Smoking, Radiotherapy, Diabetes and Osteoporosis. (PDF)**
(PDF)
