# Supplementary material for: Smoking, Radiotherapy, Diabetes and Osteoporosis as Risk Factors for Dental Implant Failure: A Meta-Analysis
Source: PLoS One. 2013 Aug 5;8(8):e71955. doi: 10.1371/journal.pone.0071955 (PMC3733795; doi:10.1371/journal.pone.0071955)

Figure A. Funnel Plot of included studies with dental implant failure risks for smoking versus non-smoking patients.

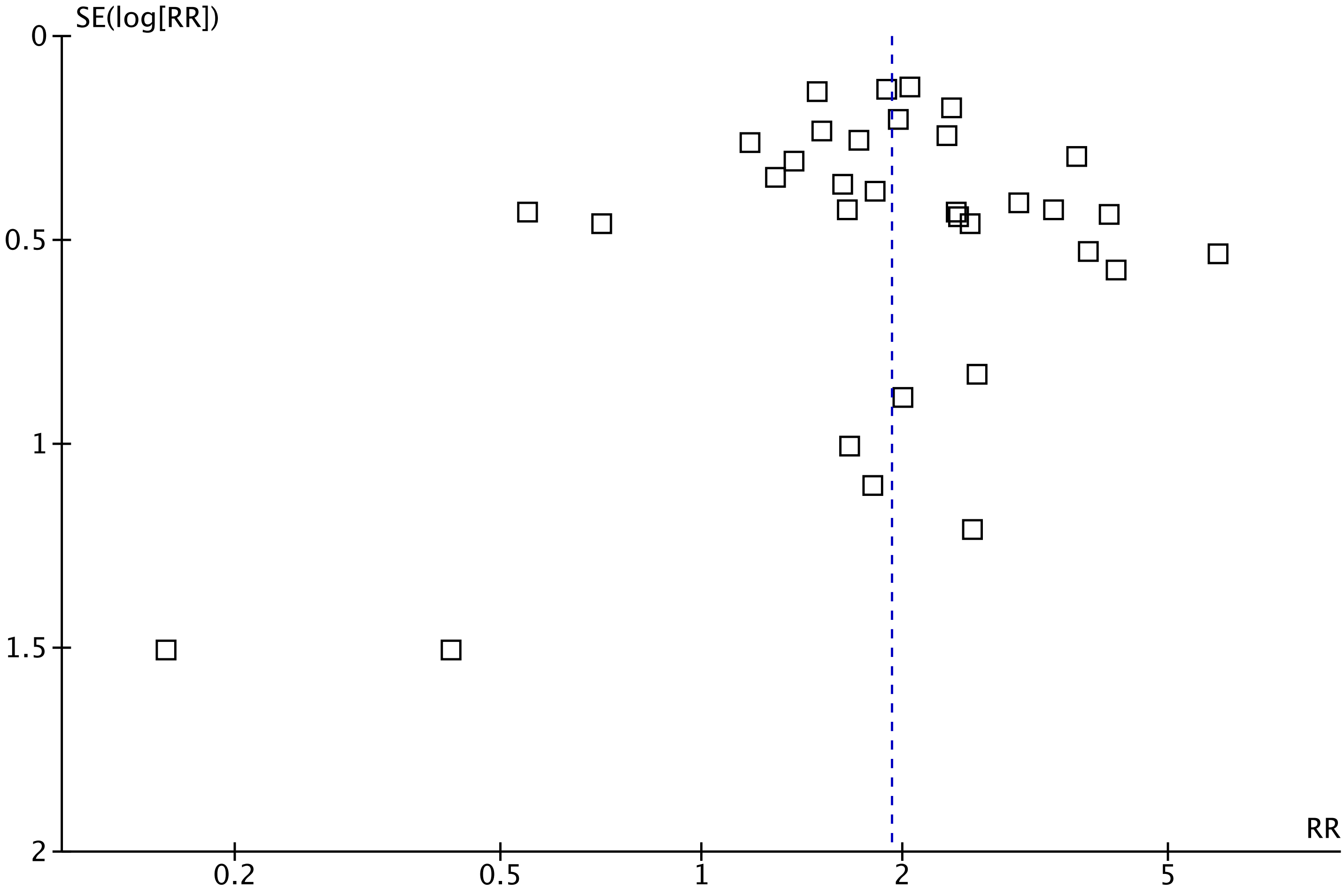

Figure B. Funnel Plot of included studies with dental implant failure risks for patients with radiotherapy versus non-radiotherapy.

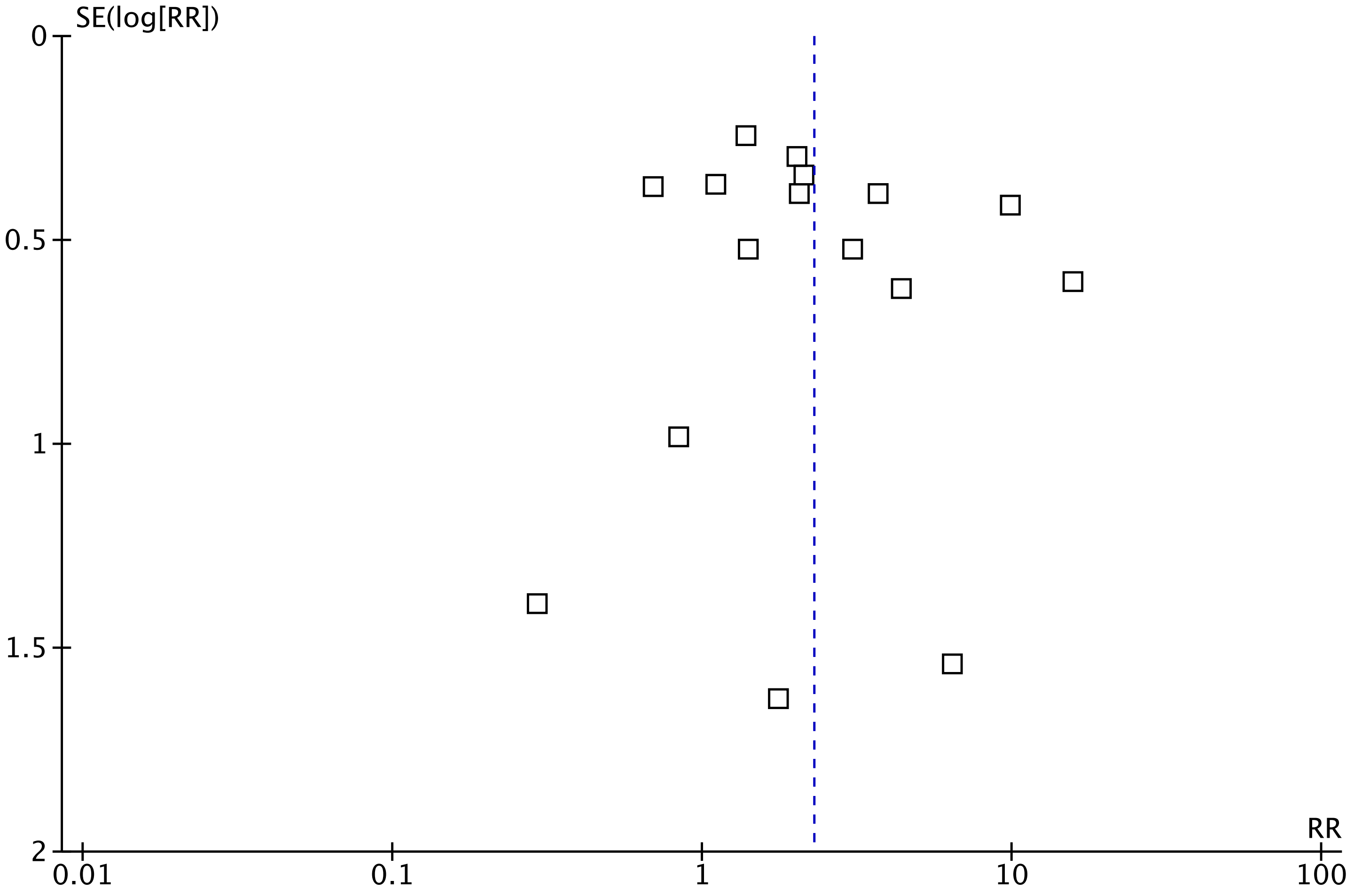

Figure C. Funnel Plot of included studies with dental implant failure risks for patients with diabetes versus non-diabetes.

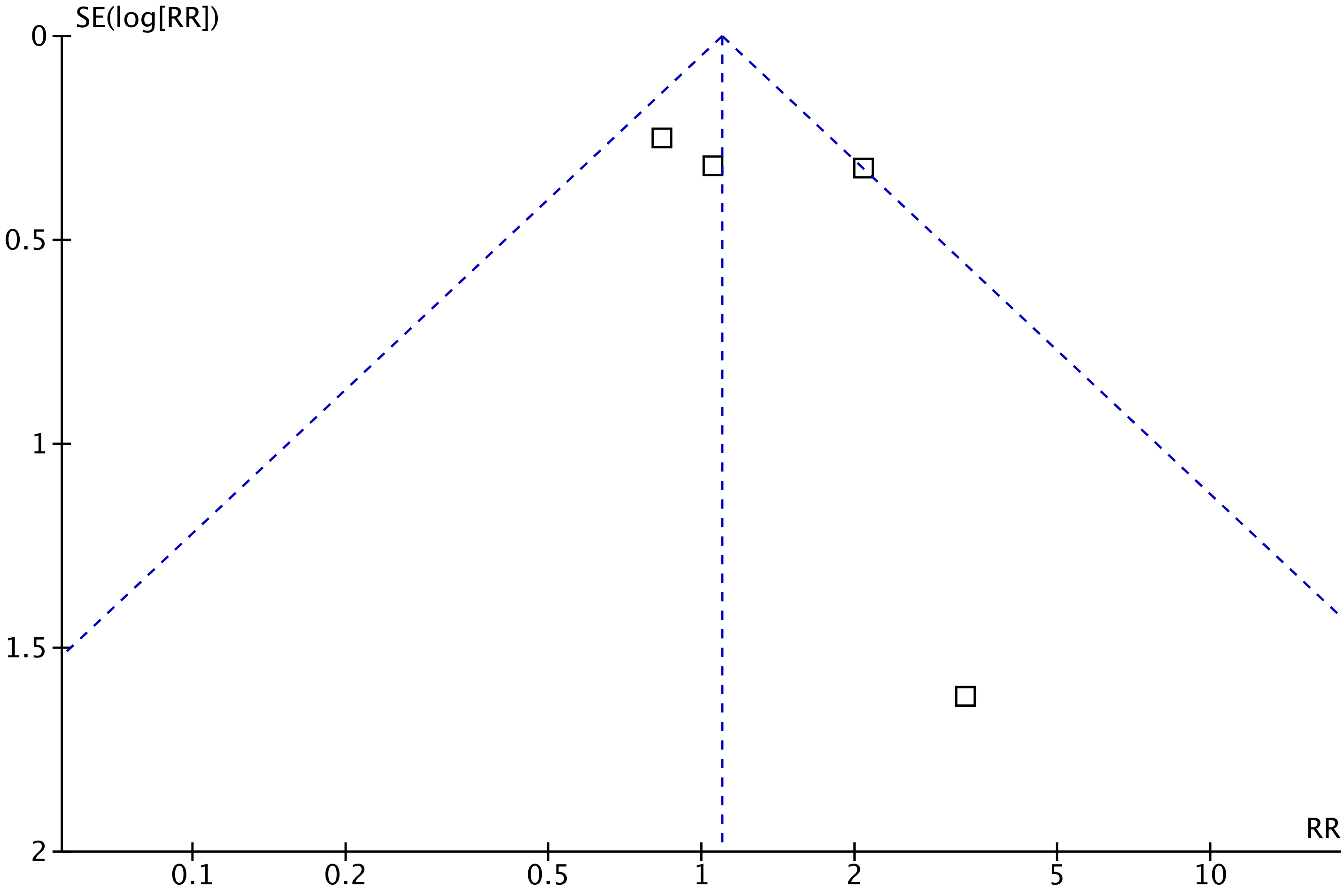

Figure D. Funnel Plot of included studies with dental implant failure risks for patients with osteoporosis versus non-oeteoporosis.

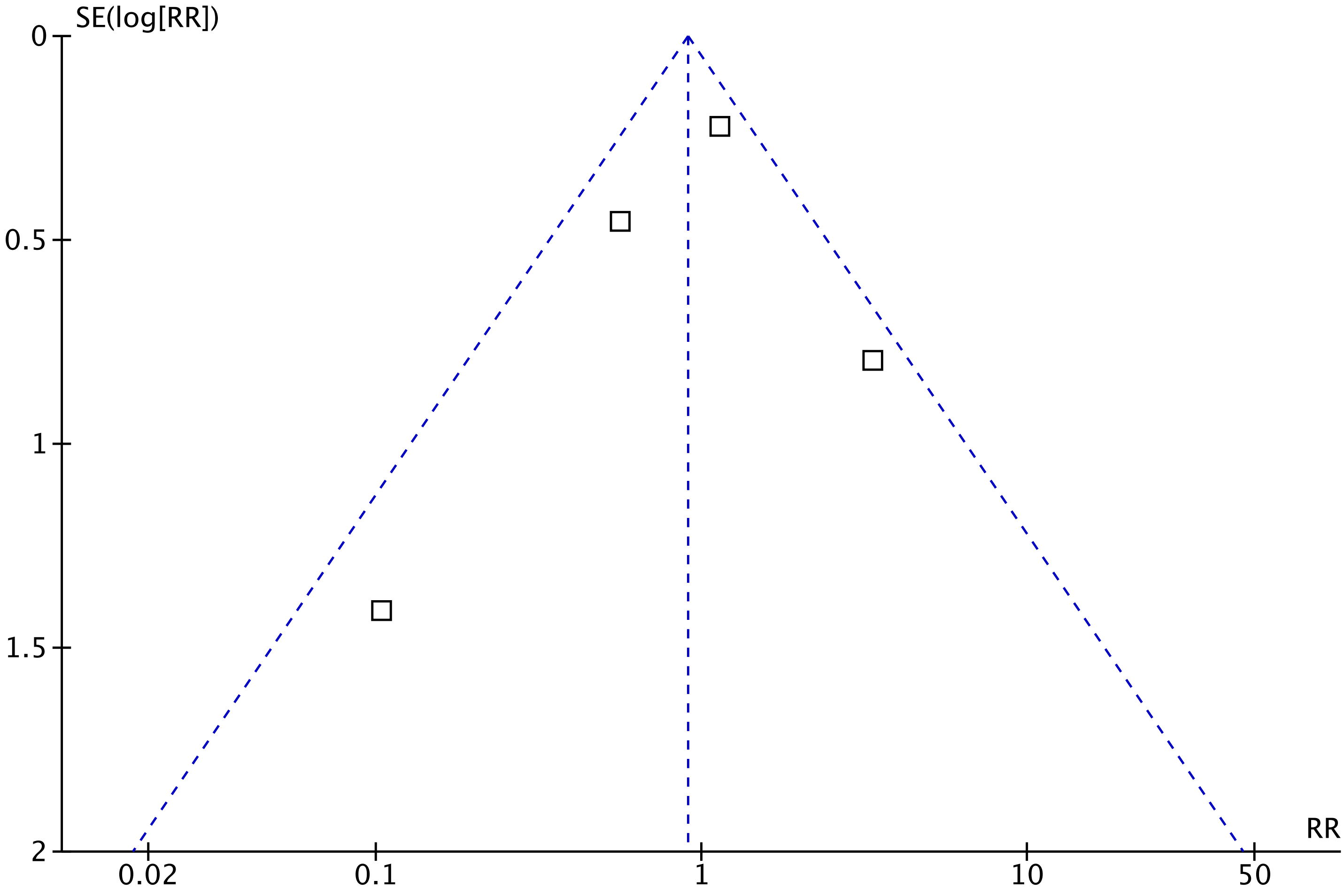

Supplement: Figure S1 — Funnel Plot of Smoking, Radiotherapy, Diabetes and Osteoporosis. (PDF) (PDF) [file pone.0071955.s002.pdf]
